# What Effect Does Epstein-Barr Virus Have on Extranodal Natural Killer/T-Cell Lymphoma Prognosis? A Review of 153 Reported Cases

**DOI:** 10.7759/cureus.17987

**Published:** 2021-09-15

**Authors:** Erika Tvedten, Jordan Richardson, Kiran Motaparthi

**Affiliations:** 1 Department of Dermatology, Michigan State University, Detroit, USA; 2 Department of Dermatology, University of Florida, Gainesville, USA

**Keywords:** enktl, epstein-barr virus, literature review, immunohistochemical, non-hodgkin lymphoma nhl

## Abstract

The primary aim of this review is to identify the relationship between Epstein-Barr virus (EBV) and prognosis in extranodal natural killer/T-cell lymphoma (ENKTL). Additionally, a literature review of ENKTL was carried out. The investigators designed and implemented a 21-year literature review using the online databases PubMed and Google Scholar. The total number of cases analyzed was 153 (64 case reports; one comparative study; one systematic review). Information related to ENKTL from July 1999 to February 2021 was included in the study. Study variables included: patient demographics, tumor classification, screening modalities, tumor characteristics, symptomatology, treatment, and prognosis. The average age at diagnosis was 50.9 years (range: 4-90 years). Patients of Asian ethnicity were most commonly affected, and there was a 1.6:1 male to female ratio. ENKTL was most frequently detected in the head and neck region, and 53.1% of cases metastasized. Of all head and neck cases, the nose was the most affected location. Immunohistochemistry positivity included: EBV (32.0%), CD2 (96.6%), CD3ϵ (81.7%), CD43 (91.7%), CD56 (86.4%), Granzyme (97.1%), Perforin (90.9%), TIA-1 (97.8%), p53 (33.3%). The most frequently employed single treatment modality was chemotherapy alone, and 34.2% of patients expired within five years of diagnosis. The average follow-up period was 16.51 months (range: 0.25-66 months). EBV was significantly associated with metastatic ENKTL (χ^2^ = 4.36; CV = 3.84; p = 0.037). We found no association between EBV and ENKTL prognosis (χ^2^ = 17.2; CV = 21.0; p = 0.14).

## Introduction and background

Extranodal natural killer/T-cell lymphoma (ENKTL) is an extremely rare subtype of non-Hodgkin lymphoma (NHL) that historically has a very low survival rate [1] and is frequently associated with Epstein-Barr virus (EBV) infection. ENKTL, a non-nasal type is a distinct clinicopathologic entity from nasal NK/T-cell lymphoma [2]. This definition was revised in 2016 by the World Health Organization (WHO) where it was determined that all cases of ENKTL were to be classified under the nasal category [3]. It is currently debated whether the diagnosis of ENKTL requires the identification of EBV infection.

Classically, ENKTL is treated with chemotherapy [4]. Radiation therapy is an essential component in the treatment of ENKTL and plays an important role in improving five-year overall survival [4,5,6].

The purpose of this paper is to answer the following clinical question, “In those subjects who have ENKTL, does EBV positivity incur a favorable prognosis?” Subject-oriented variables including demographics, imaging, diagnostic modalities, immunohistochemical profile, symptomatology, tumor location, treatment, and prognosis were investigated. The authors hypothesized that this study would reject the null hypothesis and reveal an association between EBV positivity and the prognosis of ENKTL. The aim of this study is to review the current literature concerning ENKTL as well as to elicit any relationships that exist between EBV and ENKTL.

## Review

Methods

**Figure 1:**
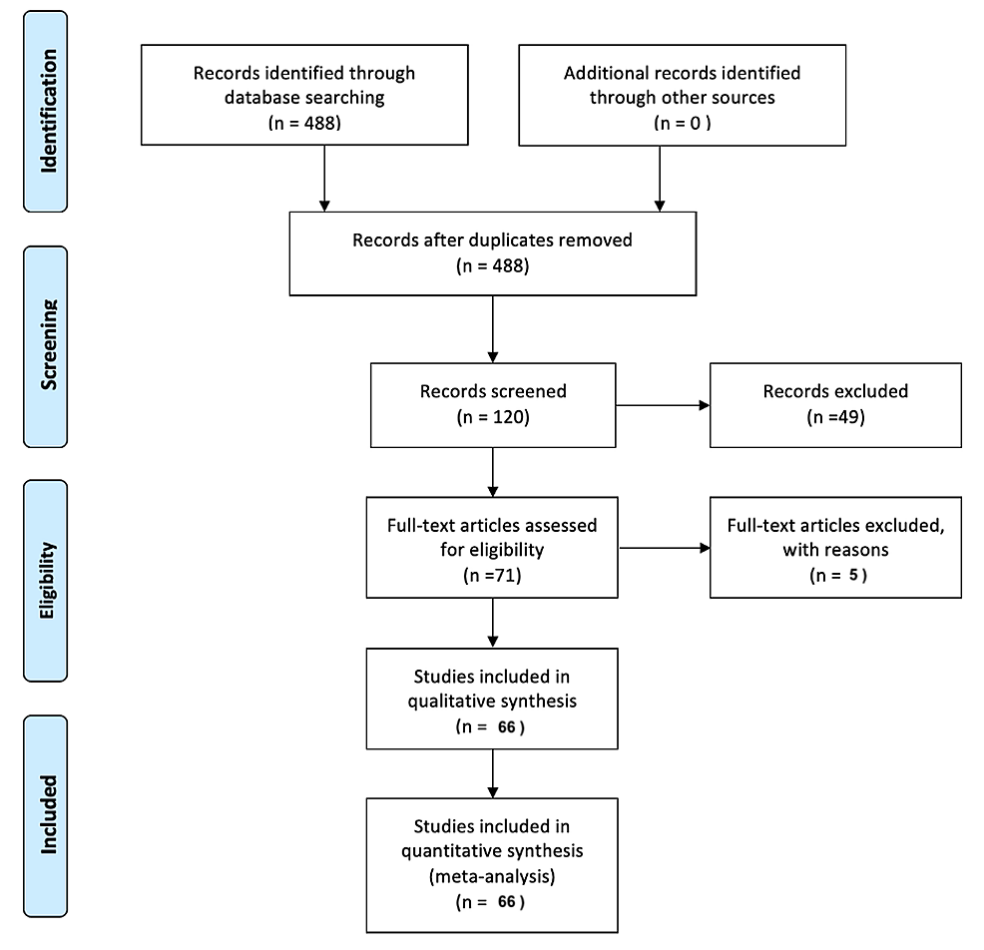
PRISM diagram. PRISM: Preferred Reporting Items for Systematic Reviews and Meta-Analyses.

Medical subject heading (MeSH) terms used for the search was “Extranodal NK/T-cell Lymphoma.” This MeSH term was used in search engines: PubMed and Google Scholar to search for articles concerning ENKTL. The Preferred Reporting Items for Systematic Reviews and Meta-Analyses (PRISMA) diagram illustrates the steps of screening and analyzing, scrutinizing the various articles during the selection process of the reports for this review (Figure 1).

Case reports, comparative studies, and systematic reviews on ENKTLs published in the English language between July 1999 and February 2021 were included. Literature that was not in the English language, not published in a peer-reviewed journal, or an animal, cadaveric, or histopathologic study without clinical information was excluded. Articles describing a previously published case were excluded to avoid double counting. The following data was collected from each study: author name, publication year, study type, patient age and gender, nasal or non-nasal classification, imaging and diagnostic modalities utilized, immunohistochemical profile, tumor location (including extension and metastasis), symptomatology, treatment, and prognosis. Data analyses were performed using Microsoft Excel 2020 version 16.35 and statistical calculations were performed using SPSS version 26 for Mac (IBM Corp., Armonk, NY, USA). Descriptive statistics were presented as mean and range for continuous variables.

Results

On the basis of inclusion criteria, 66 articles reflecting 153 cases of ENKTL were included for final review and analysis, published over the span of the past 21 years (1999-2020) [6-77]. Overall, 18.8% of the cases included came from a comparative study, 80.0% were from case reports and 1.2% were from systematic reviews. 

The mean age was 50.9 years (range: 4-90 years), with a ratio of male-to-female patients of 1.6:1. Patients of Asian descent (54.6%) were most frequently affected, followed by Caucasians (9.2%). The majority of cases were classified as the nasal type (45.8%) compared to the less common non-nasal type (18.3%) (Table 1).

**Table 1:** Extranodal NK/T-cell lymphoma: demographics, pathogenic characteristics, and treatment regimens. EBV: Epstein-Barr virus; GI: gastrointestinal; GU: genitourinary; CT: computed tomography; MRI: magnetic resonance imaging; PET: positron emission tomography; SCT: stem cell transplant.

Subject-oriented variable	N (percentage)
Gender	
Male	72 (60.0%)
Female	45 (37.5%)
Unspecified	3 (2.5%)
Ethnicity	
Asian	65 (54.6%)
Caucasian	11 (9.2%)
African American	2 (1.7%)
Hispanic	2 (1.7%)
Other	6 (5.0%)
Unspecified	33 (27.7%)
Type	
Nasal	55 (45.8%)
Non-nasal	22 (18.3%)
Unspecified	43 (35.8%)
Study Type	
Comparative Study	15 (18.8%)
Case Report	64 (80.0%)
Systematic Review	1 (1.2%)
Primary Site	
Head & Neck	57 (47.5%)
GI Tract	6 (5.0%)
Lung	1 (0.8%)
Testes	2 (1.7%)
Bone	1 (0.8%)
Skin	14 (11.7%)
Lymph Node	3 (2.5%)
Adrenal Glands	2 (1.7%)
Breast	2 (1.7%)
Soft Tissue	4 (3.3%)
Prostate Glands	1 (0.8%)
Female Reproductive Tract	2 (1.7%)
More than one site	5 (4.2%)
Miscellaneous	8 (6.7%)
Unspecified	12 (10.0%)
Head & Neck Sites	
Nose	31 (54.4%)
Nasopharynx	2 (3.5%)
Tonsil	5 (8.8%)
Hard Palate	4 (7.0%)
Tongue	1 (1.8%)
Oral Cavity	2 (3.5%)
Paranasal Sinuses	3 (5.3%)
Throat	6 (10.5%)
Parotid gland	1 (1.8%)
Jaw	1 (1.8%)
Pituitary Gland	1 (1.8%)
Metastasis	
Yes	43 (53.1%)
No	38 (46.9%)
Extension	
Yes	19 (15.8%)
No	23 (19.2%)
Unspecified	78 (65.0%)
Maximal dimension of tumor, mean (range)	6.2 cm (1.3-26.50cm)
Immunohistochemistry	
EBV	
Positive	48 (32.0%)
Negative	102 (68.0%)
CD2	
Positive	28 (96.6%)
Negative	1 (3.4%)
Cytoplasmic CD3ϵ	
Positive	76 (81.7%)
Negative	17 (18.3%)
CD43	
Positive	11 (91.7%)
Negative	1 (8.3%)
CD56	
Positive	76 (86.4%)
Negative	12 (13.6%)
Granzyme B	
Positive	33 (97.1%)
Negative	1 (2.9%)
Perforin	
Positive	10 (90.9%)
Negative	1 (9.1%)
TIA-1	
Positive	45 (97.8%)
Negative	1 (2.2%)
Presenting Symptomatology	
Respiratory alone	12 (10%)
Dermatology alone	4 (3.3%)
GI alone	2 (1.7%)
GU alone	1 (0.83%)
Neurology alone	1 (0.83%)
Systemic alone	4 (3.3%)
Swelling alone	7 (5.8%)
Symptoms involving ≥ 2 major systems	38 (31.7%)
Asymptomatic	4 (3.3%)
Miscellaneous	2 (1.7%)
Unspecified	45 (37.5%)
Imaging Modality	
Computed Tomography (CT) alone	9 (7.5%)
Magnetic Resonance Imaging (MRI) alone	1 (0.8%)
Positron Emission Tomography (PET) alone	2 (1.7%)
PET-CT alone	9 (7.5%)
Endoscopy alone	2 (1.7%)
MRI + CT	5 (4.2%)
PET-CT + CT	6 (5.0%)
Radiography + CT	1 (0.8%)
Ultrasound + PET	1 (0.8%)
Ultrasound + CT	1 (0.8%)
>2 imaging modalities	10 (8.3%)
None	4 (3.3%)
Other	1 (0.8%)
Unspecified	68 (56.7%)
Initially Misdiagnosed	
Yes	30 (25%)
No	11 (9.2%)
Unspecified	79 (65.8%)
Treatment Regimen	
Chemotherapy alone	31 (25.8%)
Radiotherapy alone	6 (5.0%)
Surgical Excision alone	1 (0.8%)
Surgical Excision + Chemotherapy	2 (1.7%)
Radiotherapy + Chemotherapy	15 (12.5%)
SCT + Chemotherapy	3 (2.5%)
Surgical Excision + Radiotherapy + Chemotherapy	5 (4.2%)
SCT + Radiotherapy + Chemotherapy	3 (2.5%)
SCT + Chemotherapy + Surgical Excision	1 (0.8%)
Supportive Treatment	2 (1.7%)
No Treatment	7 (5.8%)
Unspecified	44 (36.7%)
Outcome	
Expired - unspecified time	10 (8.3%)
Expired before or within 4 weeks of diagnosis	11 (9.2%)
Expired between 1 month and 6 months after diagnosis	20 (16.7%)
Expired between 6 months to 1 year of diagnosis	3 (2.5%)
Expired between 1 year to 5 years of diagnosis	7 (5.8%)
Alive with no evidence of disease <1 year	3 (2.5%)
Alive with no evidence of disease 1 to 5 years	25 (20.8%)
Alive with no evidence of disease for 5 years	1 (0.8%)
Alive with no evidence of disease - unspecified time	11 (9.2%)
Alive with disease for <1 year	4 (3.3%)
Alive with disease 1 to 5 years	2 (1.7%)
Lost to follow up	2 (1.7%)
Unspecified	21 (17.5%)
Follow-up period in months (range)	16.5 (0.25-66)
Age mean in years (range)	50.9 (4-90)

The majority of cases were negative for EBV (68.0%). In 53.1% of cases, the tumor metastasized. Extension to nearby structures occurred in 15.8% of tumors. Positivity was present for CD2 in 96.6% of 29 cases, CD3ϵ in 81.7% of 93 cases, CD43 in 91.7% of 12 cases, CD56 in 86.4% of 88 cases, Granzyme B in 97.1% of 34 cases, Perforin in 90.9% of 11 cases, and TIA-1 in 97.8% of 46 cases.

Stratified based on systemic origin, the most common symptoms were respiratory (10.0%) although 31.7% of cases were associated with symptoms involving ≥ two organ systems. Mere swelling was present in 5.8% of cases while 3.3% of cases were asymptomatic. The most common primary site of the tumor was within the head and neck region (47.5%). Within the head & neck, the nose was the most common specific location (54.4%) (Table 1).

The most common single imaging modality used to visualize ENKTL was evenly split between PET/CT and CT alone, both representing 7.5% of cases. PET/CT and CT combined were used in 5.0% of cases. More than two imaging modalities were utilized in ten cases (8.3%), while no imagining was utilized in four cases (3.3%) (Table 1).

Chemotherapy alone was the most commonly employed treatment modality (25.9%). Radiotherapy alone was less common (5.0%). Surgery was used alone in merely one case (0.8%) and in conjunction with other treatment modalities in eight cases (6.7%). Stem cell transplants were employed after ablation in seven cases (5.8%) (Table 1).

Regarding the outcome, slightly less than half of the prognoses were poor, as 42.5% of patients expired at follow-up. Forty patients (33.3%) were alive with no evidence of disease (ANED) while six patients (5.0%) were alive with disease (AWD). The mean follow-up period was 16.5 months, ranging from a minimum of 0.25 to a maximum of 66 months (Table 1).

A series of non-parametric chi-square tests were conducted to elicit any associations between EBV positivity and certain variables pertaining to ENKTL including metastasis, subtype (nasal vs. non-nasal), immunocompromised status, age, and prognosis. In total, 153 cases of ENKTL were extracted from 66 articles. A p-value of 0.05 was used to determine statistical significance. 

The first chi-square test is illustrated in Table 2. Out of the 153 cases of ENKTL investigated, 80 cases had information related to both EBV positivity and metastasis. The remaining 73 cases were missing at least one variable of interest. Therefore, 80 cases were included in this calculation. There was an association between EBV positivity and ENKTL metastasis (χ^2^ = 4.36; CV = 3.84; p = 0.037). 

**Table 2:** EBV positivity stratified by whether primary cancer metastasized. The Chi-square statistic is 4.36. The p-value is 0.037. The result is significant at p < 0.05. EBV: Epstein-Barr virus.

	Metastasis	No metastasis
EBV +	23	12
EBV -	19	26

Out of the 153 cases of ENKTL investigated, 76 cases had information available for both EBV positivity and subtype. Therefore, 76 cases were included in this calculation. There was no association between EBV positivity and ENKTL subtype (nasal vs. non-nasal) (χ^2^ =0.05; CV = 3.84; p = 0.82).

Out of the 153 cases of ENKTL investigated, 25 cases had information available for both EBV positivity and immunocompromised status. We defined immunocompromised status as a patient having at least one of the following conditions: cancer, HIV/AIDS, chronic corticosteroid use, chemotherapy use, autoimmune disease, organ or bone marrow transplant. Therefore, 25 cases were included in this calculation. There was no association between EBV positivity and immunocompromised status (χ^2^ =0.04; CV = 3.84; p = 0.85).

Out of the 153 cases of ENKTL investigated, 115 cases had information available for both EBV positivity and age. Age was stratified into two categories: under 35 and 35 and older. Therefore, 115 cases were included in this calculation. There was no association between EBV positivity and age (χ^2^ =0.90; CV = 3.84; p= 0.34).

Out of the 153 cases of ENKTL investigated, 59 cases had information available for both EBV positivity and prognosis. Prognosis was stratified accordingly: alive with disease <1 year, alive with disease 1-5 years, alive with no evidence of disease (unspecified time), alive with no evidence of disease <1 year, alive with no evidence of disease 1-5 years, alive with no evidence of disease 5+ years, expired (unspecified time), expired before or within four weeks of diagnosis, expired between 1-6 months of diagnosis, expired between six months and one year of diagnosis, expired between one and five years of diagnosis, lost to follow up, and unspecified. Therefore, 59 cases were included in this calculation. There was no association between EBV positivity and prognosis (χ^2^ =17.2; CV = 21.0; p = 0.14).

Discussion

The primary purpose of this literature review is to identify the relationship between EBV and prognosis in ENKTL cases. The authors hypothesized that this study would reject the null hypothesis and reveal an association between EBV positivity and the prognosis of ENKTL. A secondary aim is to review current literature involving ENKTL and to discern if any relationships exist between EBV positivity and characteristics of this disease.

The results of this study reveal no association between EBV and ENKTL prognosis (χ^2^ =17.2; CV = 21.0; p = 0.14), however, EBV was associated with ENKTL metastasis (χ^2^ =4.36; CV = 3.84; p = 0.037). 

The name NK/T-cell lymphoma was adopted to emphasize the expression of antigens associated with the most likely lineage for the malignant cells. Natural Killer cells are cytolytic cells in the primary immune response. Natural killer cells express variable T-cell lineage-associated antigens such as CD2 and CD7. Since the 1994 Pan Pacific Lymphoma Conference, the past 26 years have brought massive advancements in both understanding and treatment of this aggressive disorder that has a five-year survival rate of 25% to 50% [78]. Making a swift and accurate diagnosis of ENKTL and implementation of the standard of care treatment is crucial for enhancing the overall survival rate [4].

Extranodal NK/T-cell Lymphoma manifests in the sixth decade of life with a median age at presentation of 52 years [79]. This review identified a similar median age of onset (50.9 years). The male predilection of prior studies was also consistent with the findings of this review (1.6:1 male to females) [79-82]. While the majority of the cases (54.6%) affected Asian patients, Caucasian populations accounted for 9.2% of cases of ENKTL. 

The majority of ENKTL cases present with respiratory symptoms including nasal obstruction, epistaxis, and/or a destructive mass involving the nose, sinuses, or palate [80,81,83,84]. B-symptoms including fever, night sweats, and weight loss are present in up to 35% of patients with ENKTL [79,82]. In this review, respiratory symptoms including cough, hemoptysis, sinusitis, dyspnea, stridor, nasal congestion, pharyngitis, rhinorrhea, and dysphonia were the presenting features in 10% of reported cases. The majority of cases (31.7%) presented with symptoms pertaining to several body systems (Table 1).

The rarity and nonspecific presentation of ENKTL may delay diagnosis. In 25% of cases analyzed, the initial clinical diagnosis was erroneous, including dermatomyositis, uveitis, cellulitis, panniculitis, chronic sinusitis, pneumonia, and rhinocerebral mucormycosis.

Consistent with prior studies, the current literature review identified the head and neck region (47.5%) as the most common location for ENKTL; more specifically, the nose was the most common location for disease (54.4%). Extranodal NK/T-cell Lymphoma has metastatic potential that leads to disseminated disease and occurred in 53.1% of patients.

Definitive diagnosis of ENKTL is based on histopathology. Additionally, ENKTL demonstrates a unique immunohistochemical and genomic profile. ENKTL is characterized by a polymorphous lymphoid infiltrate that demonstrates an angioinvasive or angiodestructive pattern, invading vascular walls leading to fibrinoid and coagulative necrosis [85]. Although not quantified in this review, an angiodestructive growth pattern was visualized in the majority of cases included.

Over 90% of ENKTLs are of true NK cell origin [85]. Therefore, the most important criterion for the diagnosis of ENKTL is the demonstration of Natural Killer or T-cell markers [85]. The typical immunophenotype of ENKTL includes expression of CD2, cytoplasmic CD3 epsilon, CD56, and cytotoxic molecules such as granzyme B, TIA-1, and performing [86,87]. In the current review, 96.6%, 81.7%, and 86.4% expressed CD2, cytoplasmic CD3 epsilon, and CD56 respectively; and in regard to cytotoxic molecules granzyme B, TIA-1 and perforin were expressed 97.1%, 90.9%, and 97.8% of the time respectively (Table 1). A small number of cases of ENKTL demonstrate a clonal T-cell receptor (TCR) gene rearrangement, suggesting that tumor cells originate from cytotoxic T-cells [88].

EBV positivity as a prerequisite for diagnosis is still debated and remains controversial. Recent literature suggests that tumor location in the upper aerodigestive tract, presence of an angiocentric/angiodestructive growth pattern and necrosis, typical immunophenotype are sufficient for diagnosis of ENKTL, even if EBV infection is not identified via EBV DNA or Epstein-Barr encoding region in situ hybridization (EBER ISH) in tissue [89]. Epstein-Barr virus-negative ENKTL is a rare subtype with limited available research. In the previous series, EBV-negative ENKTL demonstrated similar clinicopathologic features compared to EBV-positive ENKTL, but EBV-negative cases occurred mainly in Chinese females, with a younger age of onset (32 years). 

Prior to the revised classification system in 2016, earlier literature reviews found a significant difference in the EBV-positivity between the nasal type and non-nasal type of ENKTL [90]. However, this relationship was not replicated in this review (χ^2^ =0.05; CV = 3.84; p = 0.82). 

Since radiation therapy is an important component of treatment for ENKTL, proper radiologic delineation of target volumes is critical [91]. In 2011, MacDonald et al. determined that Positron Emission Tomography/Computed Tomography (PET/CT) was more useful in determining the stage and volume of ENKTL compared to Computed Tomography (CT), Magnetic Resonance Imaging (MRI), and PET alone [91-93]. In this review, CT alone and PET/CT alone were tied for the most common (7.5%) imaging modality used to localize ENKTL, and > two imaging modalities were used in 8.3% of the cases analyzed (Table 1). Pretreatment imaging should include imaging of the neck, chest, abdomen, and pelvis, nasal cavity, hard palate, anterior fossa, and nasopharynx [94]. Endoscopy and bone marrow examination are also important for therapy and prognosis [95].

Therapeutic options for ENKTL are rapidly expanding, and thus, a standardized protocol regarding treatment regimens is necessary [94]. For medically fit patients with localized disease, combination modality therapy (CMT) which includes simultaneous chemotherapy and radiation is recommended. CMT is associated with superior long-term survival and local disease control with acceptable levels of adverse effects compared to chemotherapy alone [94,96].

Staging distinguishes between localized versus advanced disease. Localized disease includes ENKTL confined to the upper aerodigestive tract with limited extension into adjacent structures or cases involving cervical lymph node involvement [94]. Advanced disease includes stage three or stage four nasal type ENKTL and all stages of extranasal ENKTL, other than stage one cutaneous extranasal ENKTL [94]. An intensive asparagine-based chemotherapy regimen is associated with superior outcomes with acceptable toxicity levels compared to other chemotherapy regimens [94]. Hematopoietic cell transplantation (HCT) is used for refractory cases of ENKTL or as a post-induction consolidation therapy [94].

The prognosis of ENKTL is vastly dependent on tumor location and staging [94] and localized disease has a better prognosis than advanced disease [10,97-99]. Based on retrospective evidence, the following features represent negative prognostic factors: age >60 years, stage three or stage four disease, distant lymph node involvement, EBV positivity, and extranasal type disease [100].

Prior evidence suggests that nasal ENKTL may demonstrate a better prognosis compared to extranasal ENKTL [79]. Levels of EBV DNA in the plasma or bone marrow correlate with patient outcomes. Pretreatment plasma EBV DNA levels >500 copies/mL was associated with lower estimated three-year progression-free survival (PFS; 52 versus 79%) and overall survival (OS; 66 versus 97%) compared to lower levels of EBV DNA [101]. 

The findings in this study are not without limitations. First, only two online databases were exhausted (PubMed and Google Scholar). Chi-square analysis that was carried out in this study does not allow us to establish a cause-and-effect relationship between study variables. Moreover, for the cases that were included in the literature review, full patient charts and medical histories were not attainable. This is important as we were unable to stratify for confounding variables and this data may not be representative of the target population.

## Conclusions

The definition of ENKTL is controversial―especially when discussing EBV positivity. While some clinicians believe it is necessary for a definitive diagnosis of ENKTL, our research suggests that there is no association with patient prognosis. This project highlights the similarities between EBV positive and negative cases of ENKTL. The authors propose that the presence of an angiocentric/angiodestructive growth pattern and the typical immunophenotype with CD2, CD3ϵ, CD43, CD56, Granzyme, Perforin, and TIA-1 markers is sufficient for a presumptive diagnosis of ENKTL. The investigators recommend that clinicians pay particularly close attention to those cases of ENKTL that are positive for EBV since they are associated with metastatic potential. We encourage clinicians to continue publishing cases of ENKTL so that the conclusions drawn from this study can be expanded upon.
